# SARS coronavirus papain-like protease inhibits the type I interferon signaling pathway through interaction with the STING-TRAF3-TBK1 complex

**DOI:** 10.1007/s13238-014-0026-3

**Published:** 2014-03-14

**Authors:** Xiaojuan Chen, Xingxing Yang, Yang Zheng, Yudong Yang, Yaling Xing, Zhongbin Chen

**Affiliations:** 1 Division of Infection and Immunity, Department of Electromagnetic and Laser Biology, Beijing Institute of Radiation Medicine, Beijing, 100850 China; 2 Anhui Medical University, Hefei, 230032 China

**Keywords:** SARS coronavirus, papain-like protease, interferon, deubiquitinase, STING-TRAF3-TBK1 complex

## Abstract

SARS coronavirus (SARS-CoV) develops an antagonistic mechanism by which to evade the antiviral activities of interferon (IFN). Previous studies suggested that SARS-CoV papain-like protease (PLpro) inhibits activation of the IRF3 pathway, which would normally elicit a robust IFN response, but the mechanism(s) used by SARS PLpro to inhibit activation of the IRF3 pathway is not fully known. In this study, we uncovered a novel mechanism that may explain how SARS PLpro efficiently inhibits activation of the IRF3 pathway. We found that expression of the membrane-anchored PLpro domain (PLpro-TM) from SARS-CoV inhibits STING/TBK1/IKKε-mediated activation of type I IFNs and disrupts the phosphorylation and dimerization of IRF3, which are activated by STING and TBK1. Meanwhile, we showed that PLpro-TM physically interacts with TRAF3, TBK1, IKKε, STING, and IRF3, the key components that assemble the STING-TRAF3-TBK1 complex for activation of IFN expression. However, the interaction between the components in STING-TRAF3-TBK1 complex is disrupted by PLpro-TM. Furthermore, SARS PLpro-TM reduces the levels of ubiquitinated forms of RIG-I, STING, TRAF3, TBK1, and IRF3 in the STING-TRAF3-TBK1 complex. These results collectively point to a new mechanism used by SARS-CoV through which PLpro negatively regulates IRF3 activation by interaction with STING-TRAF3-TBK1 complex, yielding a SARS-CoV countermeasure against host innate immunity.

## Introduction

The innate immune system represents the first line of defense and initiates counteractive responses that protect the host from viral infection through evolutionarily conserved pattern recognition receptors (PRR) (Kawai and Akira, 2007). PRRs include the membrane-bound Toll-like receptors (TLRs) and cytosolic sensors, such as retinoic acid-inducible gene-I (RIG-I)-like receptors (RLRs), which sense RNA viruses (Kawai and Akira, 2007). RIG-I specifically detects the intracellular double-stranded viral RNA bearing 5′ triphosphate and panhandle structures to activate antiviral signaling (Hornung et al., 2006; Pichlmair et al., 2006). Once a host is invaded by a virus, PRRs transmit signals to the downstream kinases that activate transcription factors, including IFN regulatory factor-3 (IRF3), nuclear factor κB (NF-κB), and ATF-2/c-jun, with the help of different adaptor molecules (MAVS/IPS-1/VISA/Cardif for RIG-I, TRIF for TLR3, and MyD88 for TLR7/8/9) to activate IFN production (Barral et al., 2009; Yoneyama and Fujita, 2009; Chen and Jiang, 2013).

A previous study suggested that TRAF family members are involved in the regulation of inflammation and antiviral responses (Saha and Cheng, 2006). TRAF proteins play significant roles in signal transduction of antiviral innate immune responses. For example, TRAF3 uniquely regulates the type I IFN response and specifically contributes to TBK1-dependent IRF3 activation (Oganesyan et al., 2006; He et al., 2007). TRAF3 stimulates the non-canonical IKK-related kinase TBK1, which induces phosphorylation and dimerization of IRF3, resulting in nuclear translocation and activation of IRF3 (Fitzgerald et al., 2003). TRAF3 also interacts with several key signaling molecules, such as STING (stimulator of interferon genes, also known as MITA/ERIS/MYPS), which resides at the ER/mitochondrial membrane and forms dimers (Zhong et al., 2008; Sun et al., 2009). TRAF3, STING, and TBK1 form a signaling complex that transmits upstream sensory responses to downstream effectors and then phosphorylates IRF3 to activate type I IFN production.

Viruses have evolved elaborate mechanisms to evade or inactivate the innate immune signaling pathway for their replication (Schindler et al., 2007). Severe acute respiratory syndrome (SARS) is a highly contagious respiratory disease that appeared first in China in 2002 and has infected more than 8000 people worldwide and killed about 800 of those infected. SARS coronavirus (SARS-CoV) has a single-stranded, positive sense RNA genome of approximately 29.7 kb (Marra et al., 2003; Rota et al., 2003). Numerous studies have revealed that SARS-CoV develops an antagonistic mechanism to evade the antiviral activities of IFN (Devaraj et al., 2007; Thiel and Weber, 2008; Perlman and Netland, 2009; Zielecki et al., 2013). Several studies have shown that SARS-CoV papain-like protease (PLpro-TM) has deubiquitination (DUB) activity. PLpro-TM serves as an IFN antagonist (Sulea et al., 2005; Barretto et al., 2006; Frieman et al., 2009; Clementz et al., 2010). Previous reports have also indicated that PLpro-TM inhibits the phosphorylation and nuclear translocation of IRF3 (Devaraj et al., 2007; Frieman et al., 2009). However, there are some discrepancies in how PLpro-TM negatively regulates IRF3 activation. One study reported that PLpro-TM interacts with IRF3 (Devaraj et al., 2007). Another study rejected the finding that PLpro-TM has any influence on the phosphorylation of IRF3 (Frieman et al., 2009). A recent study has reported that PLP2 of mouse hepatitis virus A59 targets TBK1 to negatively regulate type I IFN by its DUB activity (Wang et al., 2011). It has also been reported that M protein of SARS-CoV impedes the formation of TRAF3-TBK1-IKKε complex and inhibits the production of type I IFN (Siu et al., 2009). The mechanism or mechanisms used by PLpro-TM to inhibit activation of the IRF3 pathway are not fully understood.

In the current study, we found that SARS-CoV PLpro-TM inhibits STING/TBK1/IKKε-mediated activation of type I IFN, and disrupts the phosphorylation and dimerization of IRF3. Furthermore, PLpro-TM interacts with STING, TRAF3, and TBK1, and disrupts the interaction between the components in STING-TRAF3-TBK1 complex that is required for the activation of IRF3. PLpro-TM reduces the levels of ubiquitinated forms of RIG-I, STING, TRAF3, TBK1, and IRF3, and contributes to disruption of the signaling required for the induction of IFN. Our work reveals a novel mechanism that explains how SARS PLpro efficiently inhibits activation of the IRF3 pathway, resulting in the SARS-CoV countermeasure against host innate immunity.

## Results

### PLpro-TM inhibits STING/TBK1/IKKε-mediated activation of Type I interferon

**Figure 1:**
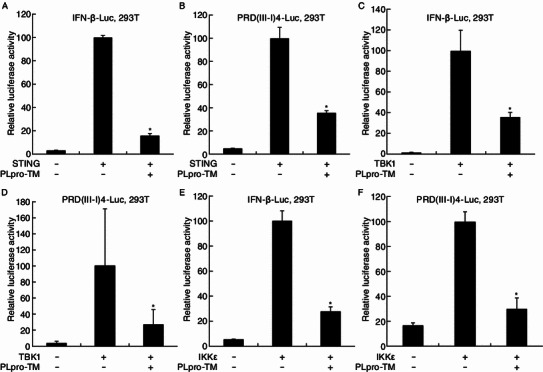
**SARS coronavirus PLpro-TM is a potent IFN antagonist**. HEK293T cells were co-transfected with either IFNβ-Luc (A, C, E) or PRD (III-I) 4-Luc (B, D, F). The plasmids expressing HA-STING (A and B), Flag-TBK1 (C and D), or Flag-IKKε (E and F) were applied to activate the IFN expression pathway and IRF3-dependent IFN expression pathway, in the presence or absence of PLpro-TM-V5. pRL-TK encoding Rellina luciferase was applied for normalization of transfection efficiency. HEK293T cells were transfected by Lipofectamine 2000 according to the manufacturer’s instructions and incubated for 24 h. The luciferase activities were assayed with the Dual Luciferase Reporter Assay. The results were expressed as mean relative luciferase activity (firefly luciferase activity divided by Renilla luciferase activity) including the standard deviation from repeated experiments carried out in triplicate. Asterisks indicate statistical significance (*P* < 0.05)

Subsequent to our previous report that SARS coronavirus PLpro negatively regulates IRF3-dependent innate immunity (Devaraj et al., 2007), we determined to address whether SARS PLpro-TM blocks STING/TBK1/IKKε-mediated activation of Type I IFN. In this study, we assessed the level of STING/TBK1/IKKε-activated IFNβ- and IRF3- promoter activities in the presence of SARS PLpro-TM. HEK293T cells were co-transfected with PLpro-TM and a combination of plasmids encoding firefly luciferase under the control of IFN-β promoter (Fig. 1A, 1C and 1E) or IRF3 promoter of PRD(III-I)4 (Fig. 1B, 1D and 1F), and STING, TBK1, or IKKε as an activator of the IFNβ signaling pathway. We found that the stimulation of HEK-293T cells with STING alone increased the activity of IFNβ- and IRF3- promoter more than 20-fold. Co-expression of STING with SARS PLpro-TM resulted in a significant decrease in IFNβ- and IRF3- promoter activities, indicating that SARS PLpro-TM can antagonize STING-mediated activation of IFNβ- and IRF3- promoter activities (Fig. 1A and 1B). SARS PLpro-TM significantly inhibited IFNβ- and IRF3- promoter activities, which are activated by TBK1 (Fig. 1C and 1D) and IKKε (Fig. 1E and 1F). These results indicate that SARS-CoV PLpro-TM antagonizes STING/TBK1/IKKε-mediated IFN-β transcription by interfering with the activation of IRF3 pathway.

### PLpro-TM antagonizes STING and TBK1-induced IRF3 activation

**Figure 2:**
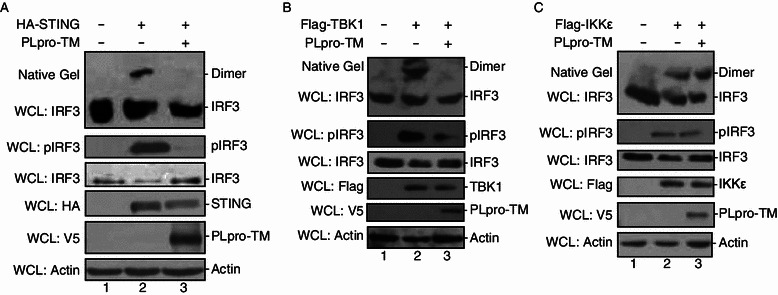
**PLpro-TM antagonizes STING and TBK1-induced IRF3 activation**. HEK293T cells were mock transfected or transfected PLpro-TM with HA-tagged STING (A), Flag-tagged TBK1 (B), or Flag-tagged IKKε (C) which was applied to activate IRF3 pathway. Proteins were extracted 24 h after transfection, the cell lysate was separated by Native PAGE followed by Western blotting to detect IRF3 monomer and dimer forms (top of Fig. 2A–C). The expression of HA-STING, Flag-TBK1, Flag-IKKε, pIRF3, IRF3, and PLpro-TM was analyzed by Western blotting performed with anti-HA, anti-Flag, anti-pIRF3, anti-IRF3, and anti-V5 antibodies, respectively. Actin was detected from whole cell lysate (WCL) as a loading control (bottom of Fig. 2A–C). Each experiment was repeated at least 3 times

Since SARS-CoV PLpro-TM antagonizes STING/TBK1/IKKε-mediated IFN-β transcription at the level of IRF3 activation, we examined whether PLpro-TM affects STING/TBK1/IKKε induced dimerization and phosphorylation of IRF3. HEK293T cells were co-transfected with HA-STING, Flag-TBK1, or Flag-IKKε in the presence or absence of V5-tagged PLpro-TM. We found that stimulation of HEK293T cells with STING alone triggered the phosphorylation and dimerization of IRF3 (Fig. 2A, lane 2). When co-transfected with PLpro-TM, IRF3 dimer and phosphorylated IRF3 were eliminated from the cells (Fig. 2A, lane 3). Overexpression of TBK1 induced dimerization and phosphorylation of IRF3 (Fig. 2B, lane 2), but PLpro-TM significantly reduced the production of phosphorylated IRF3 and completely abolished the dimerization of IRF3 (Fig. 2B, lane 3). SARS-CoV PLpro-TM did not change the amount of IRF3 dimer or phosphorylated IRF3, each of which was activated by IKKε (Fig. 2C, lanes 2 and 3). Thus, SARS-CoV PLpro-TM inhibited STING- and TBK1-mediated dimerization and phosphorylation of IRF3.

### PLpro-TM interacts with STING-TRAF3-TBK1 complex

**Figure 3:**
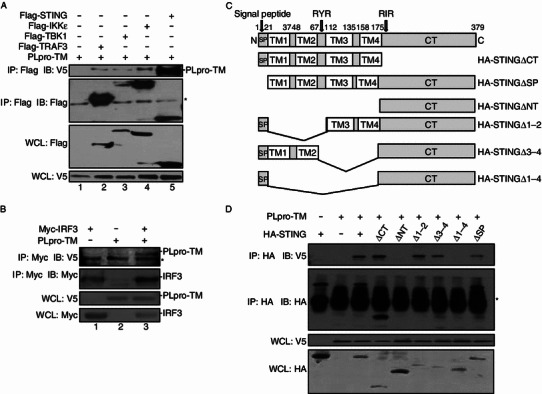
**PLpro-TM interacts with STING-TRAF3-TBK1 complex**. (A) HEK293T cells were co-transfected with expression plasmids for Flag-tagged STING, TBK1, IKKε, and TRAF3 both with and without PLpro-TM. The cell lysate was extracted 24 h after transfection. Co-immunoprecipitation experiments were performed with the indicated antibodies. The co-immunoprecipitation product was blotted by using anti-V5 (top panel), and re-probed by using anti-Flag (second panel) to detect TRAF3, TBK1, IKKε, and STING. The input lysate was blotted by using anti-Flag to detect TRAF3, TBK1, IKKε, STING (third panel), and anti-V5 to detect PLpro-TM (bottom panel). The asterisk indicates the nonspecific band. (B) HEK293T cells were transfected with Myc-tagged IRF3 in the absence and in the presence of PLpro-TM. The cell lysate was extracted 24 h after transfection. Co-immunoprecipitation was performed with the indicated antibodies. The co-immunoprecipitation product was blotted by using anti-V5 (top panel), and re-probed by using anti-Myc (second panel) to detect IRF3. The input lysate was blotted using anti-V5 to detect PLpro-TM (third panel), and anti-Myc to detect IRF3 (bottom panel). The asterisk indicates the nonspecific band. (C) Diagrams of full-length and truncated STING proteins used in the study. TM, transmembrane domain. CT, C-terminus domain. (D) The association of PLpro-TM with HA-STING or its truncated mutants. HEK293T cells were co-transfected with plasmids expressing HA-tagged STING or its truncated mutants, with or without PLpro-TM. The cell lysate was extracted 24 h after transfection. Co-immunoprecipitation was performed with the indicated antibodies. The co-immunoprecipitation product was blotted by using anti-V5 (top panel), and re-probed using anti-HA (second panel) to detect STING and its truncated mutants. The input lysate was blotted by using anti-V5 to detect PLpro-TM (third panel), and anti-HA to detect STING and its truncated mutants (bottom panel). The asterisk indicates the nonspecific band. Every experiment was repeated for at least 3 times

Because SARS PLpro-TM inhibited STING- and TBK1-dependent activation of the IRF3 pathway, PLpro-TM might associate with STING-TRAF3-TBK1 complex, either directly or as part of the multiprotein complex. To confirm this hypothesis, we examined whether PLpro-TM interacted with the key signaling components in the complex. Twenty-four hours after transfection of HEK293T cells with PLpro-TM and Flag-STING, TRAF3, TBK1, or IKKε, the interactions between PLpro-TM and these regulating proteins were assessed via co-immunoprecipitation and Western blotting. The interactions between PLpro-TM and STING, TRAF3, TBK1, and IKKε were confirmed (Fig. 3A). The association between PLpro-TM and IRF3 was also identified (Fig. 3B). These results indicate that PLpro-TM interacts with the STING-TRAF3-TBK1 complex.

Both PLpro-TM and STING are membrane-bound proteins that are anchored to the endoplasmic reticulum (ER) membrane with the transmembrane (TM) domain (Huang et al., 2012; Ouyang et al., 2012; Shang et al., 2012; Shu et al., 2012; Yin et al., 2012), and the TM domains at the N-terminus of STING were found to be indispensable for IRF3 activation *in vitro* (Tanaka and Chen, 2012). Our recent study demonstrated that coronavirus PLpros negatively regulate antiviral innate immune response by disruption of STING-mediated signaling (Sun et al., 2012). In this study, we further addressed which STING domains are required for the interaction between STING and PLpro-TM. We constructed a series of deletion mutants of STING, as described in Fig. 3C. The association of STING mutants with PLpro-TM was assessed by co-transfection of PLpro-TM and each of the STING mutants into HEK293T cells. We found that the association between STING and PLpro-TM was completely abolished when 4 TM domains at the N-terminus of STING were deleted (Fig. 3D, lanes 5 and 8). Partial deletion of TM domains or the C-terminal helicase domain did not affect the interaction between STING and PLpro-TM (Fig. 3D, lanes 4, 6, and 7). These findings indicate that PLpro-TM interacts with STING through the TM domains.

### PLpro-TM disrupts STING-TRAF3-TBK1 interaction

**Figure 4:**
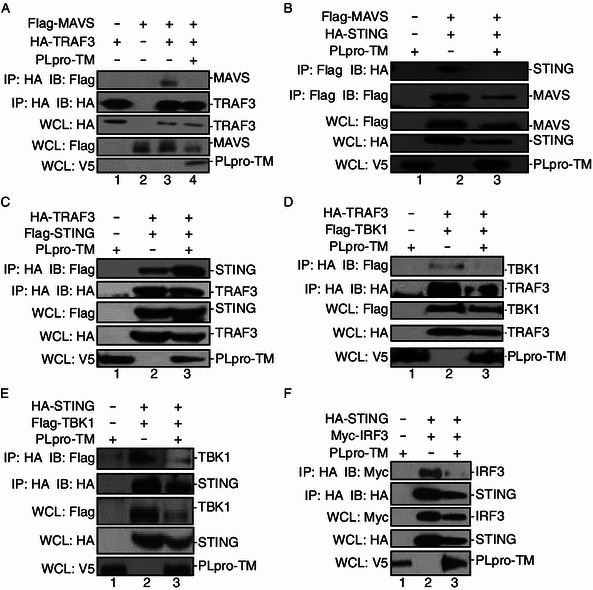
**PLpro-TM disrupts the formation of STING-TRAF3-TBK1 complex**. (A, B) HEK293T cells were transfected with Flag-MAVS together with either HA-tagged TRAF3 (A) or HA-tagged STING (B) with or without PLpro-TM. Twenty-four hours after transfection, the cell lysate was collected, and subjected to immunoprecipitation (IP) and immunoblotting (IB) using the indicated antibodies. Co-immunoprecipitation was blotted using anti-Flag or anti-HA (Fig. 4A and 4B, top panels), and re-probed using anti-HA or anti-Flag (Fig. 4A and 4B, second panels) for HA-TRAF3 (Fig. 4A) or Flag-MAVS (Fig. 4B). The input lysate was blotted using anti-Flag or anti-HA to detect MAVS (Fig. 4A, third panel) or STING (Fig. 4B, third panel), and anti-V5 to detect PLpro-TM (Fig. 4A and 4B, bottom panels). (C and D) HEK293T cells were co-transfected with HA-TRAF3 together with either Flag-tagged STING (C) or Flag-tagged TBK1 (D) and PLpro-TM. Twenty-four hours after transfection, the cell lysate was prepared and subjected to immunoprecipitation (IP) and immunoblotting (IB) with the indicated antibodies. (E and F) HA-STING and Flag-tagged TBK1 (E) or Myc-tagged IRF3 (F) were co-transfected into HEK293T cells with or without PLpro-TM. Twenty-four hours after transfection, the cell lysate was collected and subjected to immunoprecipitation (IP) and immunoblotting (IB) with the indicated antibodies. Each experiment was repeated at least 3 times

Because PLpro-TM interacts with the components of the STING-TRAF3-TBK1 complex, we hypothesized that PLpro-TM would impede the formation of functional STING-TRAF3-TBK1 tripartite complex. To test this hypothesis, we assessed the impacts of PLpro-TM on the assembly of the STING-TRAF3-TBK1 complex. DNA plasmids expressing TRAF3, MAVS, STING, and TBK1 were co-transfected into HEK293T cells in the absence and in the presence of PLpro-TM. The cell lysate was co-immunoprecipitated (Fig. 4). We observed that co-immunoprecipitation of TRAF3 with MAVS or TBK1 was disrupted in the presence of PLpro-TM (Fig. 4A, lane 4 and Fig. 4D, lane 3). The assembly of STING with MAVS was obviously disrupted by PLpro-TM (Fig. 4B, lane 3). We also found that PLpro-TM disrupted the co-immunoprecipitation of STING with IRF3 (Fig. 4F, lane 3). However, the expression of PLpro-TM had no effect on co-immunoprecipitation of TRAF3 and STING (Fig. 4C, lane 3), and the interaction of STING with TBK1 (Fig. 4E, lane 3). Thus, SARS-CoV PLpro-TM disrupted the interaction between the key components of STING-TRAF3-TBK1 complex, which explains how PLpro-TM suppresses the activation and nuclear translocation of IRF3.

### PLpro-TM blocks ubiquitination of STING-TRAF3-TBK1 complex

**Figure 5:**
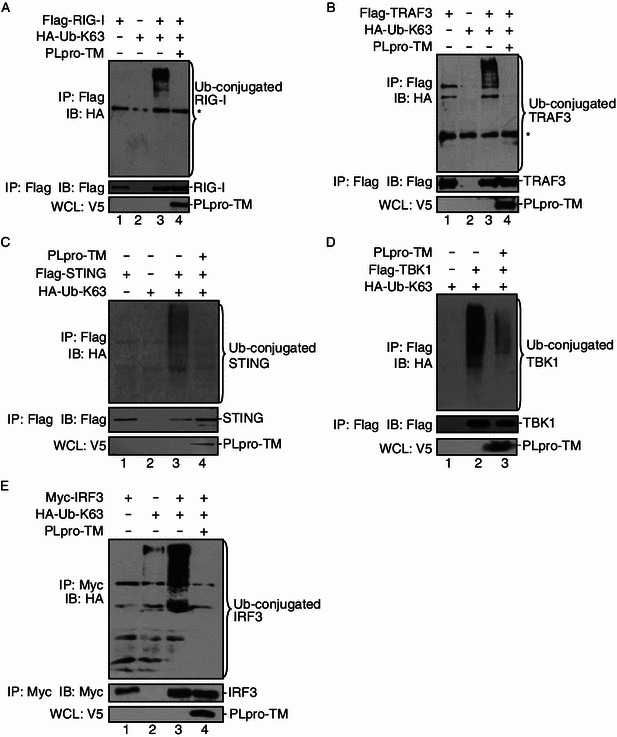
**PLpro-TM reduces the ubiquitination levels of signaling molecules**. The plasmids expressing Flag-tagged RIG-I (A), TRAF3 (B), STING (C), TBK1 (D), and Myc-tagged IRF3 (E) together with HA-Ub-K63 were co-transfected into HEK293T cells in the presence and in the absence of V5-tagged PLpro-TM. Cells were incubated for 24 h after transfection and treated with 25 μmol/L MG132 for 4 h prior to the harvest of the cell lysate. The cell lysate was immunoprecipitated with the indicated antibodies. The products were immunoblotted using anti-HA to evaluate K63-linked ubiquitinated proteins (upper panels). The whole cell lysate (WCL) was blotted to evaluate the expression of PLpro-TM (bottom panels). Each experiment was repeated at least 3 times. The asterisk indicates the nonspecific band from cellular proteins with high affinity with Agarose

Multiple regulatory molecules located upstream of IRF3 in the IFN pathway require ubiquitination, especially K63-linked ubiquitination, and deubiquitination, which play critical roles in the activation of IFN responses (Bibeau-Poirier and Servant, 2008; Bhoj and Chen, 2009; Isaacson and Ploegh, 2009; Zhong et al., 2010). Our team and others have previously reported that SARS PLpro-TM has DUB activity, which acts as a negative regulator of the innate immune response (Barretto et al., 2005; Lindner et al., 2005; Sulea et al., 2005; Barretto et al., 2006; Ratia et al., 2006; Devaraj et al., 2007; Chen et al., 2009; Frieman et al., 2009; Clementz et al., 2010; Sun et al., 2012). In the current study, we examined whether SARS-CoV PLpro-TM could recognize and deubiquitinate the key signaling molecules in the IFN signaling pathway. HEK293T cells were co-transfected with HA-Ub-K63 and plasmids which encode RIG-I, TRAF3, STING, TBK1, or IRF3. The cell lysate was immunoprecipitated to verify the ubiquitination status of those immunoprecipitated proteins. We found reductions at the levels of poly-ubiquitinated RIG-I (Fig. 5A), TRAF3 (Fig. 5B), STING (Fig. 5C), TBK1 (Fig. 5D), and IRF3 (Fig. 5E) in cells expressing PLpro-TM. These findings indicated that PLpro-TM inhibited the ubiquitination of RIG-I, TRAF3, STING, TBK1, and IRF3 through its DUB activity. As ubiquitination of these signaling regulators is required in the formation of STING-TRAF3-TBK1, this result is in agreement with the finding that SARS-CoV PLpro-TM blocks activation of IFN by disrupting the formation of STING-TRAF3-TBK1 mediated complex.

## Discussion

**Figure 6:**
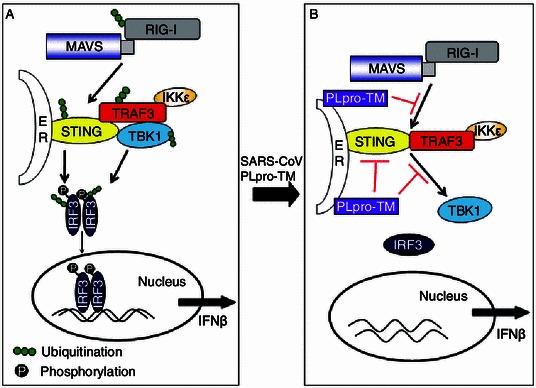
**A hypothetical model depicting the mechanisms used by SARS-CoV PLpro-TM to disrupt the STING-TRAF3-TBK1 complex required for activation of the IFN-β production pathway**. (A) Viral infections are sensed by pattern-recognition receptors (PRRs), which in turn recognize pathogen-associated molecular patterns (PAMPs) and trigger antiviral responses. RIG-I identifies the 5′-3p RNA, and then interacts with MAVS to activate the IFN responses factor 3 (IRF3). In MAMs (mitochondrial-associated membranes), MAVS also interacts with STING that locates at the ER (endoplasmic reticulum), and induces the ubiquitination and dimerization of STING. The activated STING recruits TBK1 and IRF3 and contributes to the phosphorylation of IRF3 mediated by TBK1. STING recruits TBK1 and IKKε and forms the TBK1-IKKε complex via the association with TRAF3. The TBK1 complex induces the phosphorylation, dimerization, and nuclear translocation of IRF3. (B) When SARS-CoV invades the host, these processes are disrupted by SARS-CoV PLpro-TM. PLpro-TM disrupts activation of the IRF3 pathway induced by STING-TRAF3-TBK1 by blocking the assembly of the complex, and it deubiquitinates the signaling molecules RIG-I, TRAF3, STING, TBK1, and IRF3

SARS-CoV PLpro (PLpro-TM) acts as an IFN antagonist that reduces the production of IFN by inhibiting activation of the IRF3 pathway (Devaraj et al., 2007; Frieman et al., 2009; Clementz et al., 2010; Sun et al., 2012), but the underlying mechanisms used by SARS PLpro-TM to inhibit activation of IRF3 pathway are not fully understood. In this study, we uncovered a novel mechanism used by SARS PLpro-TM to inhibit activation of the IRF3 pathway. We found that (1) SARS-CoV PLpro-TM inhibits the activation of transcription factor IRF3 and negatively modulates the IFNβ signaling pathways, which results from PLpro-TM impeding STING- and TBK1-activated phosphorylation and dimerization of IRF3. (2) PLpro-TM interacts with TRAF3, TBK1, IKKε, STING, and IRF3, the key components that assemble a regulator complex for activation of IFN expression. However, the interaction between the components in STING-TRAF3-TBK1 complex is disrupted by PLpro-TM. (3) SARS PLpro-TM reduces the levels of ubiquitinated forms of RIG-I, STING, TRAF3, TBK1, and IRF3, the key components in the STING-TRAF3-TBK1 complex. These results revealed a novel mechanism used by SARS coronavirus to negatively regulate activation of innate immunity (Fig. 6).

The most striking finding of this study is that PLpro-TM disrupts the STING-TRAF3-TBK1 complex, which leads to inhibiting IRF3 activation. STING is important in regulating the production of IFN (Ishikawa and Barber, 2008). STING binds to both TBK1 and IRF3 and is necessary for the activation of IRF3 (Tanaka and Chen, 2012). TRAF3 locates at ER-to-Golgi transport compartments. Co-localization of TRAF3 and STING allows them to be involved in the induction of type I IFN innate immunity (van Zuylen et al., 2012). TRAF3 binds to TBK1 and forms a complex with TBK1, linking upstream signaling responses of RIG-I/Mda5-MAVS-STING to the TBK1-directed phosphorylation of IRF3 and IFNβ transcription (Fig. 6A) (Fitzgerald et al., 2003; Oganesyan et al., 2006). In this study, we showed that SARS-CoV PLpro-TM interacts with the components of STING-TRAF3-TBK1 complex, and that PLpro-TM inhibits formation of the STING-TRAF3-TBK1 complex that is necessary for IFNβ transcription. PLpro-TM also disrupts the interaction between these key signaling proteins and prevents STING-TRAF3-TBK1 complex from activating IRF3 (Fig. 6B). We confirm that SARS PLpro-TM interacts with STING, a recently identified regulator that facilitates the recruitment of TBK1. This is in agreement with our recent finding that NL63 CoV PLP2 disrupts the dimerization of STING and deubiquitinates STING for modulation of IFN expression (Sun et al., 2012). Along with our previous findings, this study revealed a new mechanism that PLpro-TM interacts with STING by disruption of the STING-TRAF3-TBK1 complex, which leads to inhibiting IRF3 activation. Our findings suggest that SARS coronavirus PLpro-TM interacts with STING through the TM domain at the N-terminus of STING. STING forms a membrane trafficking system that mediates the dynamic translocation of STING and leads to the effective induction of innate immune responses (Saitoh et al., 2010), but the interaction between PLpro-TM and STING prohibits the membrane trafficking of STING, which results in inhibition of phosphorylation and dimerization of IRF3 and blocks IFN signaling transduction (Fig. 6B).

Ubiquitination and deubiquitination emerge as the key mechanisms that regulate the virus-induced type I IFN signaling pathways (Bibeau-Poirier and Servant, 2008; Bhoj and Chen, 2009; Isaacson and Ploegh, 2009; Zhong et al., 2010). Ubiquitination, especially K63-linked ubiquitination, is critically involved in regulation of the activation of related receptors, such as RIG-I, and transduction of cell signaling pathways in innate immune responses (Zeng et al., 2010). In contrast, deubiquitination inhibits the activation of signaling molecules, and cellular and viral DUBs play important roles in negative regulation of host innate immunity. A20, named after its cDNA clone No. and also referred to as tumor necrosis factor α-induced protein (TNFAIP) 3, has DUB activity that is a central gatekeeper in inflammation and immunity. It removes K-63 linked poly-ubiquitin chains from RIP1, TRAF6, RIP2, and NEMO and negatively regulates the innate immune responses (Coornaert et al., 2009). Cellular protein DUBA specifically deubiquitinates TRAF3 that is critical for activation of IRF3, resulting in inhibiting phosphorylation of IRF3 (Kayagaki et al., 2007; Wang et al., 2011). Recent studies revealed that coronaviruses, including human CoV NL63, MHV, and SARS-CoV, have evolved to encode DUBs for modulating the innate immunity (Barretto et al., 2005; Sulea et al., 2005; Chen et al., 2007; Frieman et al., 2009; Clementz et al., 2010; Wang et al., 2011; Sun et al., 2012). We have reported that human CoV NL63 reduces the ubiquitinated forms of STING, RIG-I, TBK1, and IRF3 to negatively regulate IFN signaling (Sun et al., 2012). MHV-A59 PLP2 distinctively targets and deubiquitinates TBK1 to negatively regulate cellular type I IFN signaling pathway (Wang et al., 2011). DUBs have been reported in other viral proteins, such as arterivirus EAV PLP2 (van Kasteren et al., 2013), herpes simplex virus type 1 (HSV-1) UL36 (Kattenhorn et al., 2005), and porcine reproductive and respiratory syndrome virus (PRRSV) nsp2 (Chen et al., 2010; Sun et al., 2010). These viral proteins act as IFN antagonists through the DUB activity.

Multiple SARS coronaviral proteins, such as ORF3b, ORF6, N, M, and nsp1, have been suggested to inhibit IFN production (Kamitani et al., 2006; Devaraj et al., 2007; Frieman et al., 2007; Kopecky-Bromberg et al., 2007; Wathelet et al., 2007; Zust et al., 2007; Narayanan et al., 2008; Siu et al., 2009). SARS M protein is associated with TRAF3, which is crucial for the IFN responses and impairs the function of TRAF3-TBK1-IKKε (Siu et al., 2009). The formation of TRAF3-TBK1 complex is an essential step in the activation of IRF3 (Siu et al., 2009). We found in thestudy that when TRAF3 and TBK1 were co-expressed in HEK293T cells, the interaction between TRAF3 and TBK1 was observed in cells. However, the interaction between TRAF3 and TBK1 was completely disrupted in the presence of PLpro-TM. Furthermore, the interaction between STING and TBK1 was significantly decreased when coexpressed with PLpro-TM. These data collectively indicate that PLpro-TM impeded the interactions between the components of STING-TRAF3-TBK1 complex, which results in inhibition of the activation of IRF3. There are differences between M protein and PLpro-TM in the regulation of IRF3 activation. SARS M protein inhibits the activation of IRF3 mediated by both TBK1 and IKKε (Siu et al., 2009). When IKKε and PLpro-TM were co-expressed in HEK293T cells, we found that SARS-CoV PLpro-TM did not inhibit IKKε-mediated phosphorylation and dimerization of IRF3. However, IKKε activation of the IRF3-dependent IFNβ-Luc and PRD (III-I)4-Luc reporters is obviously inhibited by PLpro-TM. This discrepancy may be due to one or more other mechanisms explaining how PLpro blocks IKKε-mediated ubiquitination and translocation of IRF3. Additionally, the Gn cytoplasmic tail of Hantavirus forms a complex with TRAF3 that disrupts the formation of TBK1-TRAF3 complexes and their downstream signaling responses required for IFNβ transcription (Alff et al., 2008; Matthys and Mackow, 2012). This may be a common mechanism used by human viruses to regulate antiviral innate immunity by preventing formation of the STING-TRAF3-TBK1 complex.

In summary, our findings uncover a new mechanism used by SARS-CoV through which PLpro-TM negatively regulates IFN activation, as depicted in Fig. 6. SARS PLpro-TM blocks: 1) STING and TBK1-medaited phosphorylation and dimerization of IRF3; 2) the STING-TRAF3-TBK1 complex formation; and 3) the ubiquitination of the key signaling molecules in the IFNβ expression pathway, such as STING, TRAF3, TBK1, and IRF3. Our study has yielded strong evidence of the mechanisms underlying SARS-CoV countermeasures against host innate immunity.

## Materials and methods

### Cell culture, transfection reagent, and antibodies

HEK 293T cells were cultured in Dulbecco’s Modified Eagle’s Medium (GIBCO) supplemented with 10% heat-inactivated fetal bovine serum (FBS) and maintained at 37°C in a humidified 5% CO_2_ incubator. Transient transfection reagent Lipofectamine 2000 was purchased from Invitrogen. Anti-HA (MBL), anti-Flag (MBL), anti-V5 (MBL), anti-Myc (MBL), anti-β-Actin (Beyotime), anti-IRF3 (Abcam), and anti-pIRF3 (Abcam) antibodies were used in the study.

### Plasmid DNAs

The wild-type SARS-CoV PLpro-TM, which containing PLpro core domain (aa 1541 to 1855 and the TM domain (aa 2139–2425), and plasmids of IFN-β-Luc, PRD (III-I) 4-Luc, and HA-tagged Ub-K63 were previously described (Devaraj et al., 2007; Clementz et al., 2010). Constructs of Flag-MAVS, Myc-IRF3, Flag-TBK1, Flag-RIG-I, and Flag-RIG-IN were kindly provided by Dr. Himanshu Kuma and Shizuo Akira (Immunology Frontier Research Center, Osaka University, Osaka, Japan). Flag-STING, HA-STING, and Flag-IKKε were kindly provided by Dr. Zhengfan Jiang (School of Life Sciences, Peking University, Beijing, China) and Dr. Xuemin Zhang (National Biomedicine Center, Beijing). HA-TRAF3 and Flag-TRAF3 plasmids were kindly supplied by Dr. Kui Li (University of Tennessee, Memphis, Tennessee, USA).

### Construction of STING deletion mutants

A series of STING mutants were constructed as shown in Fig. 3C. Briefly, STING mutants were amplified by PCR or overlap PCR from HA-STING construct, and inserted into pCMV-HA vector with a HA tag at the N-terminus of each STING mutant. The forward (P_1_) primer and reverse primer (P_2_) for each mutant were as follows: STINGΔCT P_1_: 5′-CGGAATTCCGCCCCACTCCAGCCTGCATCCATCCC-3′, STINGΔCT P_2_: 5′-CCGCTCAGAGTCAGAGCTCTGGCAGGATCAGCCG-3′. STINGΔSP P_1_: 5′-CGGAATTCCGGCAGCCTTGGTTCTGCTGAGTGCC-3′, STINGΔSP P_2_: 5′-CCGCTCGAGTCAAGAGAAATCCGTGCGGAGAGG-3′. STINGΔNT P_1_: 5′-CGGAATTCCGCAGGCCCGGATTCGAACTTACATTC-3′, STINGΔNT P_2_: 5′-CCGCTCGAGTCAAGAGAAATCCGTGCGGAGAGG-3′. STINGΔ1–2 P_1_: 5′-GTGAAGGGCGGGCCGACCGCCTTCTGGGCCCCGTGACCCC-3′, STINGΔ1–2 P_2_: 5′-GCGGTCGGCCCGCCCTTCAC-3′. STINGΔ3–4 P_1_: 5′-TAAGTTCGAATCCGGGCCTGAGCCAGGCTGCAGACCCCGTTTAA C-3′, STINGΔ3–4 P_2_: 5′-CAGGCCCGGATTCGAACTTAC-3′. STINGΔ1–4 P_1_: 5′-TAAGTTCGAATCCGGGCCTGCTTCTGGGCCCCGTGACCCC-3′, Δ1–4 P_2_: 5′-CAGGCCCGGATTCGAACTTAC-3′. PCRs were performed with LA Taq (TaKaRa) for the initial denaturation at 94°C for 5 min, then 30 cycles of 94°C for 30 s, 55°C for 30 s, and 72°C for 60 s, and extension at 72°C for 60 s. For some of the STING mutants, overlap PCR was performed. During the first-round PCR, 2 small fragments were amplified with the use of HA-STING as a template for the initial denaturation at 94°C for 5 min, then 30 cycles of 94°C for 30 s, 55°C for 30 s, and 72°C for 60 s, and extensions at 72°C for 10 min. The amplicons were purified and fused together by 5 cycles of 94°C for 30 s, 55°C for 30 s, and 72°C for 60 s, and extensions at 72°C for 10 min, followed by 25 cycles of 94°C for 30 s, 55°C for 30 s, and 72°C for 60 s, and extensions at 72°C for 10 min. All the STING deletion mutants were cloned into pCMV-HA vector and confirmed by endonuclease digestion analysis and DNA sequencing.

### Luciferase assay

HEK293T cells were transfected with the reporter plasmid DNAs (pRL-TK, IFN-β-Luc, or PRD (III-I) 4-Luc, the IRF3 promoter controlled Luciferase reporter, and SARS PLpro-TM) using Lipofectamine 2000 according to the manufacturer’s protocol and incubated for 24 h. Firefly luciferase and Renilla luciferase activities were assayed using the Dual Luciferase Reporter Assay Kit (Promega). The results were expressed as mean values of the relative luciferase activity (firefly luciferase activity divided by Renilla luciferase activity) including the standard deviation from repeated experiments carried out in triplicate. For statistical analysis, differences between the values of regulators and regulators co-transfected with PLpro-TM were subjected to unpaired, 2-tailed Student’s *t* test performed with the use of Microsoft SPSS 12.0 software. A *P* value of <0.05 was considered statistically significant (Vaux et al., 2012).

### Western blotting analysis

HEK 293T cells were seeded in 60-mm-diameter dishes and incubated at 37°C for 18 h. The cells were subsequently transfected with SARS PLpro-TM or empty vector using Lipofectamine 2000 reagent according to the manufacturer’s instruction. Twenty-four hours after transfection, cells were lysed in RIPA buffer, containing protease inhibitor cocktail at 4°C for 30 min, spun down at 12,000 rpm for 10 min. Protein extracts in 40 μL of 2× SDS-PAGE sample buffer were boiled for 10 min. Samples were separated on SDS-PAGE gel and transferred to NC membrane. Blots were incubated with indicated primary antibodies. After 3 washings in 1× TBS-T buffer, blots were incubated with HRP-conjugated secondary antibodies (Beyotime, China). Antibody-antigen reactions were detected with the use of Western Lighting Plus-ECL chemiluminescence reagents (Biomed, China). Each experiment was repeated at least 3 times.

### IRF3 dimer detection

To assess IRF3 dimerization, HEK293T cells were transfected with HA-STING or Flag-TBK1/IKKε (1 μg) in the presence and in the absence of V5-tagged PLpro-TM (1 μg). Twenty-four hours after transfection, the cell lysate was fractionated by 8% Native Gel in running buffer containing 1% sodium deoxycholate at 4°C, as previously described (Frieman et al., 2009). After electrophoresis, proteins were transferred and analyzed by Western blotting with the indicated antibodies. Each experiment was repeated at least 3 times.

### Co-immunoprecipitation (Co-IP) analysis

The effects of SARS PLpro-TM in disrupting the interaction between STING and associated proteins in cultured cells were assessed as previously described (Gack et al., 2007). Briefly, HEK293T cells were co-transfected with Flag/HA-STING (2 μg), Flag-TRAF3 (2 μg), Flag-TBK1/IKKε/MAVS (2 μg), and Myc-IRF3 (2 μg) in the presence and in the absence of V5-tagged PLpro-TM (2 μg) with the use of Lipofectamine 2000. Twenty-four hours later, cells were lysed in RIPA buffer (50 mmol/L Tris-HCl pH 7.4, 150 mmol/L NaCl, 2 mmol/L EDTA, 1% NP-40) containing protease inhibitor cocktail (1 mmol/L, Roche), 0.1% SDS, and 10 μmol/L NEM at 4°C for 30 min. The cell extracts were then spun down at 12,000 rpm for 10 min at 4°C. The soluble lysate was immunoprecipitated with anti-Flag/HA antibody for at least 12 h, the lysate was then precleared by the addition of 40 μL of protein A + G agrose (Beyotime) and rocked at 4°C for 6 h. This was followed by spinning down the beads. The bead-antibody-protein complex was spun down and washed 4 times with 1 mL RIPA buffer or PBS. The protein was eluted from the beads in 40 μL of 2× SDS-PAGE sample buffer subjected to boiling for 10 min. The proteins were separated by SDS-PAGE and transferred to NC membrane for Western blotting. Each experiment was repeated at least 3 times.

### Assessing ubiquitination of signaling molecules

The effect of SARS PLpro-TM on ubiquitinated proteins in cultured cells was assessed as previously described (Evans et al., 2004; Clementz et al., 2010). Briefly, Flag-tagged RIG-I, TRAF3, STING, TBK1, IKKε, and Myc-tagged IRF3 (1.0 μg each) were co-transfected into HEK293T cells cultured in 60-mm dishes together with pcDNA3.1-HA-Ub-K63. Transfection was performed with Lipofectamine 2000 according to the manufacturer’s instructions. Empty vector pcDNA3.1/V5-HisB was used to standardize the total amount of DNA used for transfection. After 24 h, cells were incubated with 25 μmol/L MG132 for 4 h, then harvested by the addition of 300 μL RIPA buffer (50 mmol/L Tris-HCl pH 7.4, 150 mmol/L NaCl, 2 mmol/L EDTA, 1% NP-40) containing protease inhibitor cocktail (1 mmol/L, Roche), 0.1% SDS, and 10 μmol/L NEM at 4°C for 30 min, spun down at 12,000 rpm for 10 min. Non-covalently bound proteins were dissociated by boiling the lysate in 1% SDS; samples were diluted 1:10 in lysis buffer (50 mmol/L Tris-HCl pH 7.4, 150 mmol/L NaCl, 2 mmol/L EDTA, 1% NP-40) containing protease inhibitor cocktail and 10 μmol/L NEM. The soluble lysate was then immunoprecipitated with anti-Flag/HA antibody for at least 12 h, the lysate was then precleared by adding 40 μL protein A + G agrose (Beyotime) and rocked at 4°C for 6 h, followed by spinning down the beads. The beads-antibody-protein complex was spun down and washed 4 times with 1 mL RIPA buffer or PBS. The protein was eluted from the beads in 40 μL of 2× SDS-PAGE sample buffer subjected to boiling for 10 min. The samples were separated by SDS-PAGE and transferred to NC membrane for Western blotting. Each experiment was repeated at least 3 times.

## Abbreviations

DUB: deubiquitination
EAV: equine arteritis virus
ER: endoplasmic reticulum
HSV-1: herpes simplex virus type 1
IFN: interferon
IKKε: IkappaB kinase
IRF3: IFN regulatory factor-3
MHV: mouse hepatitis virus
MAVS: mitochondria antiviral signaling protein
NEMO: NF-κB essential modulator
NF-κB: nuclear factor κB
ORF: open reading frames
PLpro: papain-like protease
PRR: pattern recognition receptors
PRRSV: porcine reproductive and respiratory syndrome virus
RIP: receptor interacting protein
RLRs: retinoic acid-inducible gene-I (RIG-I)-like receptors
SARS-CoV: severe acute respiratory syndrome coronavirus
STING: stimulator of interferon genes
TBK1: TANK-binding kinase 1
TLRs: Toll-like receptors
TM: transmembrane
PLpro-TM: the membrane-anchored PLpro domain
TRAF: tumor necrosis factor receptor-associated factor
PRD: positive regulatory domain
